# Effects of an online program including mindfulness, exercise therapy and patient education compared to online exercise therapy and patient education for people with Patellofemoral Pain: protocol for a randomized clinical trial

**DOI:** 10.1186/s12891-023-06491-x

**Published:** 2023-05-11

**Authors:** Liliam Barbuglio Del Priore, Vitoria Ozores Perez, Ronaldo Valdir Briani, Lucca Andre Liporoni Bego Farinelli, Júlia de Cássia Pinto da Silva, Odisséia Marli Gimenes Martins, Fábio Arruda Lopes, Anita Barros Amorim, Evangelos Pappas, Fábio Mícolis de Azevedo

**Affiliations:** 1 Department of Physiotherapy, School of Science and Technology, Sao Paulo State University (UNESP), 305 Roberto Simonsen St, Presidente Prudente, SP 19060-900 Brazil; 2 Brazilian Center for Mindfulness and Health Promotion - Mente Aberta, Federal University of Sao Paulo (Unifesp), Sao Paulo, Brazil; 3 Department of Computer Engineering, Virtual University of Sao Paulo State (UNIVESP), Dracena, Brazil; 4 School of Health Sciences, Faculty of Medicine and Health, The University of Sydney, Sydney, Australia; 5 School of Medicine and Illawarra Medical and Health Research Institute, University of Wollongong, Wollongong, Australia

**Keywords:** Anterior knee pain, Psychological factors, Mindfulness, Education, Exercise therapy

## Abstract

**Background:**

Patellofemoral pain (PFP) is a common knee disorder that causes persistent pain, lower self-reported function and quality of life. People with PFP also present with altered psychological factors, which are associated with higher levels of pain and dysfunction. Mindfulness-based interventions (MBI) generally consist of meditative practices developed to provide a holistic approach to chronic conditions. However, the effects of MBI on clinical and psychological outcomes for people with PFP remains understudied.

**Methods:**

This assessor-blinded, parallel, two-arm randomized clinical trial aims to investigate the effects of adding an 8-week online MBI program to exercise therapy and patient education on clinical and psychological factors for people with PFP. We also aim to investigate whether psychological factors mediate changes in pain and function. Sixty-two participants with PFP will be recruited and randomized into one of two treatment groups (Mindfulness or Control group). Both groups will receive an 8-week intervention involving exercise therapy and education delivered through an online platform. The Mindfulness group will additionally receive a MBI component including formal and informal practices. Outcomes will be assessed online at baseline, intervention endpoint (follow-up 1) and 12 months after intervention completion (follow-up 2). Comparisons between groups will be performed at all time points with linear mixed models. A mediation analysis will be performed using a 3-variable framework.

**Discussion:**

Exercise therapy and patient education are considered the “best management” options for PFP. However, unsatisfactory long-term prognosis remains an issue. It is known that people with PFP present with altered psychological factors, which should be considered during the evaluation and treatment of people with PFP. Adding a MBI to the current best treatment for PFP may improve short and long-term effects by addressing the underlying psychological factors.

**Trial registration:**

*Registro Brasileiro de Ensaios Clínicos* (ReBEC) RBR-4yhbqwk, registered in April 6, 2021.

**Supplementary Information:**

The online version contains supplementary material available at 10.1186/s12891-023-06491-x.

## Introduction

Patellofemoral pain (PFP) is characterized by the presence of pain around or behind the patella, exacerbated by activities that increase patellofemoral joint loading [1]. PFP is prevalent in young adults and adolescents (22.7% and 28.9%, respectively), with women being twice as likely to develop PFP than men [2]. People with PFP typically report reduced levels of physical activity [3], functional capacity [4, 5], and quality of life [6]. Evidence also indicates that symptoms can be long-lasting, with 50 to 91% of people with PFP experiencing persistent pain up to 18 years after the initial diagnosis [7].

According to the International Association for the Study of Pain (IASP) [8], the concept of pain encompasses more than just physical-chemical aspects of nociception. Sociocultural, emotional, and cognitive factors can also contribute to the worsening of pain and dysfunction [9–13]. This seems to apply to people with PFP as they present with altered psychological factors such as anxiety, depression, pain catastrophizing, and kinesiophobia [11]. Moderate correlations between psychological factors with pain and disability have also been reported in people with PFP [14].

Current recommendations on the “best management” for people with PFP are exercise therapy and patient education [15, 16]. Although effective in the short-term [16], unsatisfactory long-term prognosis remains an issue, with 57% of the people with PFP reporting unfavorable recovery at 5–8 years [17]. This could be a reflection of the lack of consideration for psychological factors during rehabilitation. Doménech et al. [18] have reported that patients who experience the largest decreases in pain catastrophizing, kinesiophobia, anxiety, and depression also experience greater improvement in pain and disability after a purely biomedical treatment. It has been suggested that the addition of co-interventions to address psychological factors, for example, cognitive-behavioral treatment, reassurance, and graded exposure to activity may enhance rehabilitation outcomes, such as pain and function, in individuals with PFP [18, 19]. The specific mechanisms by which changes in psychological factors influence physical function are not well known, however, positive coping cognitions and emotional states are thought to confer resilience to pain and resourcefulness to improve adherence to active treatments and physical activity [18, 19]. Therefore, further investigation is required to understand the potential additional effects of interventions that may influence psychological factors to the current best management of PFP.

Mindfulness-based interventions (MBI) were developed to assist people in managing stress, anxiety and chronic pain [20]. This evidence-based program has been increasingly used for a variety of musculoskeletal disorders [21, 22]. Mindfulness is defined as a form of bringing attention, friendly curiosity, and non-judgmental awareness to body sensations, thoughts, and emotions in order to reduce suffering or distress and to increase wellbeing [23]. Previous studies have demonstrated specific brain modifications in neuroimaging evaluation in experience practitioners, such as increased grey matter volume in the frontal lobe and relatively decreased posterior cingulate cortex activity compared to novice practitioners [24–26]. This finding suggests an existence of a neural network responsible for the positive effects of MBI practices including, but not restricted to information processing, mind wondering regulation and adaptative behavior [24]. Therefore, as part of a rehabilitation program, mindfulness may promote a better focus on rehabilitation [20] and influence several psychological factors such as anxiety, pain catastrophizing and avoidance behaviors [27–29].

In this context, a recent study [30] has reported that adding an MBI to exercise therapy promoted lower levels of pain during running and stepping, less functional limitations and lower pain catastrophizing as compared to exercise alone in female runners with PFP. However, this study was performed exclusively on female recreational runners with PFP, which limits the generalizability to the general population with PFP. In addition, patient education was not provided in this study, which is of utmost importance to PFP [31]. As such, more studies are needed to investigate the effects of MBI in addition to exercise therapy and patient education in people with PFP.

Internet-based interventions have been recently promoted due to their potential to overcome geographical barriers, increase access to health services, and provide alternative means to continue treating patients whenever face-to-face encounters are precluded [32]. There is evidence supporting the use of internet-based interventions for the treatment of several conditions [33, 34] as they may provide similar improvements in pain and function compared to face-to-face treatments [35, 36]. Furthermore, internet-based interventions may allow patients to assume a more active role in their rehabilitation, encouraging strategies as self-management and self-efficacy [33]. Online MBI has also been shown to be feasible and effective in reducing psychological factors such as stress, anxiety and depression [37]. However, few studies have investigated the effects of internet-based interventions for PFP, especially including components targeting psychological factors such as MBI.

The aims of this randomized clinical trial are:

(i) to investigate the immediate (8-week) and long-term (12-month) effects of adding the MBI program to an 8-week online intervention comprised of exercise therapy and patient education on self-reported recovery, pain, function, and psychological factors in people with PFP;
(ii) to investigate whether changes in psychological factors mediate changes in pain and function.

We hypothesize that people in the Mindfulness group will experience greater decreases in pain, as well as higher improvements in function at 8 weeks and 12 months. We also hypothesize that psychological factors such as kinesiophobia and pain catastrophizing will mediate changes in pain and function.

## Methods

### Protocol elaboration

This protocol is reported according to the SPIRIT statement (Standard Protocol Items: Recommendations for Interventional Trials) [38] and CONSORT Statement [39].

### Study design

This is an assessor-blinded, parallel, two-arm randomized clinical trial with 12-month follow-up. All participants will receive an identical internet-based exercise therapy and patient education intervention, with one group receiving additional online MBI program. Details of participants time schedule according to the SPIRIT recommendations are available in Additional file 1.

### Participants and consent

People with knee pain will be recruited through social media to participate in this study. All participants who meet the eligibility criteria will be informed about the nature of the research and receive an online consent form, prepared in accordance with the declaration of Helsinki [40] and the 466/12 resolution of the National Health Council.

### Eligibility criteria

The eligibility criteria were designed according to the most recent PFP consensus statement on clinical examination of PFP [1] and will be completed through an online form. Participants’ eligibility will be confirmed by a physiotherapist with > 3 years of clinical experience managing people with PFP. All assessments, including eligibility criteria and outcomes measures (baseline, follow-up 1 and follow-up 2), will be performed through online forms. No face-to-face physical examinations will be performed. However, if further details are required to confirm the diagnosis, an online meeting between the physiotherapist and the participant will be performed.

### Inclusion criteria

Participants will be required to meet the following criteria in order to be included in this study: (i) age between 18 and 40 years old; (ii) self-reported anterior knee pain (unilateral or bilateral) when performing at least two of the following activities: prolonged sitting, squatting, kneeling, running, ascending and descending stairs, jumping and landing [1]; (iii) self-reported anterior knee pain with insidious onset lasting at least 6 months [41]; (iv) worst self-reported pain in the previous month corresponding to at least 30 mm in a 100 mm visual analogue scale (VAS) [42].

### Exclusion criteria

Participants will be excluded if they meet any of the following criteria: (i) self-reported anterior knee pain caused by trauma on the knee; (ii) self-reported history of patellar dislocation or subluxation; (iii) self-reported history of meniscal injury, ligament instability or patellar tendinopathy; (iv) history of osteoarthritis in any lower limb joint; (v) history of surgery on any lower limb joint; (vi) patient-reported rheumatic or neurologic disease; (vii) physiotherapy treatment for PFP during the preceding 6 months; (viii) answer “yes” on any questions on the PAR-Q physical activity readiness questionnaire [43]; (ix) history of current or past psychosis, major depressive episode, suicide attempt, post-traumatic stress disorder, bipolar disorder, manic episode, or substance dependency.

### Randomization and blinding

The randomization list will be developed by an investigator who will not be involved in the recruitment and assessment of the participants. Randomization codes will be generated in blocks, using a custom list on the website (https://www.sealedenvelope.com/), and the participants will be randomized with a 1:1 allocation [44] to one of the two interventions. Sealed opaque envelopes, sequentially enumerated, will be used to conceal the allocation. After the baseline assessment, the investigator will open the envelope containing the participant’s random code to ensure the allocation of the participant will be concealed. Due to the nature of the interventions, participants will be informed about the type of intervention. Therefore, the study cannot be considered double-blind [44]. The assessor will be blinded to the allocation of participants.

### Outcome measures

Outcome measures will be assessed online at baseline, intervention endpoint (8 weeks – follow-up 1), and 12 months after intervention completion (follow-up 2). Demographic data (e.g. age, gender, duration of symptoms) will be recorded at the baseline assessment.

#### Primary outcomes

##### Self-reported recovery

The 7-point Likert global rating of change scale (GROC) is a measure of treatment effect that has been previously used in people with PFP [45, 46]. The participants will be asked “How would you describe your knee pain now, compared to before you began the treatment?” The answers are marked on a 7-point Likert scale (much better, better, a little better, no change, a little worse, worse, much worse). The answers will be dichotomized in “successful” and “unsuccessful”. A successful outcome will be defined as being much better or better.

##### Pain

Participants’ self-reported pain level over the previous week will be measured with a 100 mm VAS [42]. The VAS consists of a 0 to 100 mm horizontal line, with 0 representing “no pain” (0 mm) and 100 representing “extreme pain”. Participants will be instructed to draw a perpendicular line on the scale at the position that indicates the severity of usual and worst knee pain over the preceding week. The VAS is valid and reliable for assessing people with PFP [42].

#### Secondary outcomes

##### Self-reported function

The Anterior Knee Pain Scale (AKPS) is a valid and reliable 13-item questionnaire that evaluates subjective function related to PFP [42]. Participants will complete the AKPS based on their perceived knee condition at the prior week. The total score for the AKPS ranges from 0 (maximal disability) to 100 (no disability), with the total score being used for statistical analysis.

##### Anxiety and depression

The Hospital Anxiety and Depression Scale (HADS) is a 14-item questionnaire that evaluates the emotional state of the patient and identifies cases of mild, moderate and severe anxiety and/or depression disorders [47]. The HADS consists of two subscales, which assess anxiety (7 items) and depression (7 items) separately. Participants will be asked to answer each item on a 4-point Likert scale (0–3), with scores ranging from 0 to 21 for anxiety and 0 to 21 for depression. Scores between 0 and 7 are classified as normal, between 8 and 10 as mild, between 11 and 14 as moderate, and between 15 and 21 as severe [48].

##### Kinesiophobia

The Tampa scale for kinesiophobia is a self-administered questionnaire that assesses pain-related fear associated with the avoidance behaviors, movements and physical activity [49, 50]. It contains 17 statements with answers in a 4-point Likert scale: [1] Strongly disagree, [2] Partially disagree, [3] Partially agree and [4] Totally agree[50]. Participants will be instructed to choose the option according to how much they agree with each statement. The score ranges from 17 to 68 and the higher the score, the higher the fear [49, 51].

##### Pain catastrophizing

The Pain Catastrophizing Scale (PCS) is a 13-item questionnaire that consists of describing thoughts and feelings that individuals experience when they have pain [52]. Participants will be instructed to reflect on the experiences caused by pain in the past and indicate their perception on a 5-point Likert scale, where (0) represents “not at all” and [4] “all the time”. The higher the score, the greater the pain catastrophizing [53].

##### Pain self-efficacy

The Chronic Pain Self-Efficacy Scale (CPSS) is a 22-item self-administered questionnaire that assesses the perception of self-efficacy and the ability to deal with the consequences of pain in patients with chronic pain [54]. The CPSS contains 3 domains: pain control, physical function and symptom control. Participants will be asked to answer how much they agree with each of the items arranged on a Likert scale ranging from 10 to 100 points. The score ranges from 30 to 300, where the higher the score, the better the self-efficacy.

##### Self-reported physical activity level

The International Physical Activity Questionnaire short form is a 9-item questionnaire that assesses how many days and hours the participants usually spent per week doing several activities [55]. The physical activity level will be determined by the total of vigorous and moderate exercise in the previous week and calculated according to previous studies [55, 56].

### Interventions

After the baseline assessment, participants will receive immediate access to a WEB platform developed by one of the authors available at http://www.stepslab.com.br/ where the interventions will be delivered. An individualized online meeting will be performed between participants and a physiotherapist not involved in data analysis to guide them regarding platform usage, deadlines and the importance of committing to the intervention. Participants will have access to the online interventions for 8 weeks, which will be immediately ceased at the end of the period. The exercise and education contents will be developed and pre-recorded by two physiotherapists with more than three years of clinical experience using evidence-based material. The MBI content will be developed and pre-recorded by a certified mindfulness teacher with more than 15 years of experience and revised by a psychologist to ensure psychological appropriateness. Details of the interventions according to the TIDier checklist [57] are available in Additional file 2.

Participants will only have access to content related to the group to which they were allocated (restricted area). Each session will be released for access on pre-defined dates relative to participants’ entry into the trial (immediately after baseline assessment). This will be performed by an investigator who will not be involved in the recruitment and assessment of the participants. The system will only allow opening of the next session if the participant had ended the previous one. At the end of each session, participants will be required to report their current level of pain on an online VAS scale, if there was any adverse event during or after the intervention, the level of satisfaction and the level of perceived exertion on a 15-point Borg scale [58], ranging from 6 (no exertion at all) to 20 perceiving a (maximal exertion).

**Fig. 1:**
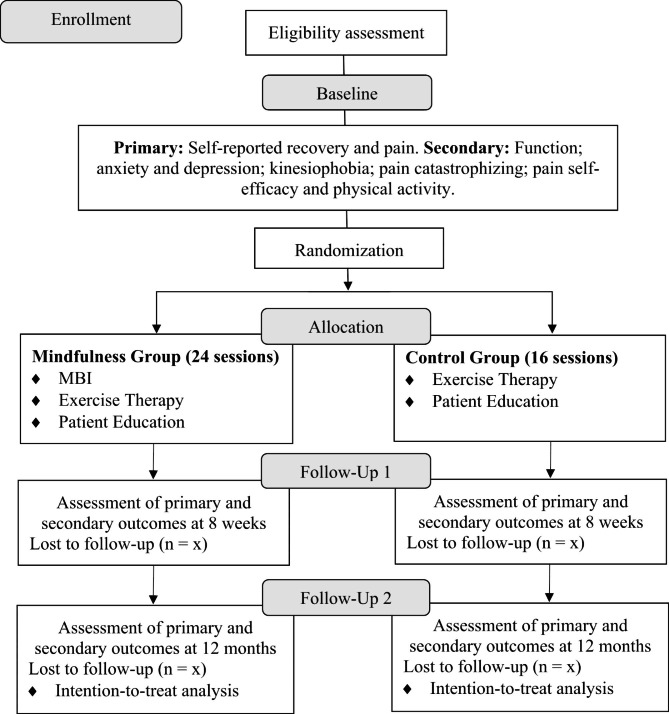
Flow diagram of the study

Participants will be instructed to not seek any other kind of knee pain treatment during the study, except in emergency cases. If necessary, participants will be able to contact the therapist through the platform’s e-mail. An outline of the study procedures is summarized in Fig. 1.

#### Control group

Participants allocated to this group will receive two pre-recorded video classes per week according to their availability (e.g. on Tuesdays and Thursdays) with Exercise therapy and Patient education contents (lasting 35–50 min in total).

##### Exercise therapy

The exercise therapy component will include the prescription of the exercises according to the American College of Sports Medicine Position Statement [59] and the PFP consensus [16, 19]. The mean duration of the exercise videos will be approximately 30 min. There will be a one-day break between the sessions, to respect recovery time.

Exercise therapy will aim to improve muscle performance, movement coordination and mobility [16, 19]. This intervention will target hip, knee, and ankle muscles. Exercises will be progressed in phases every two weeks (i.e., intensity, type of exercise, technique or repetitions). During the exercise, affirmative and encouraging audio messages will be displayed in order to motivate the participant to finish the session with as much effort as possible. The intensity of the exercises will be monitored through the Borg scale and must remain between 12 and 16 points. If the participant report exertion values outside this range, the exercise intensity will be modified. The full description of the exercises is available in Additional file 3.

##### Patient education

Educational pre-recorded video classes will cover the following topics.

- Week 1: Understanding my knee: anatomy and biomechanics of the knee and the relationship between pain and injury.
- Week 2: Understanding my knee pain: incidence and prevalence of PFP; why my knee hurts; biomechanical and psychological factors of PFP; prognosis and diagnosis of PFP.
- Week 3: “Too Much, too soon”: how high volume or high load intensity during daily activities or sports can lead to knee pain.
- Week 4: Myths and truths about my knee: Knee crepitus and movements considered harmful to the joint; fear of movement; and imaging exams.
- Week 5: Aspects of quality of life that influence pain: sleep quality, weight control, confidence, coping strategies, and mental health.
- Week 6: Taking care of my own pain: self-management of pain, motivation and responsibility for your own health.
- Week 7: Available treatment options: importance of adherence to active treatments, treatments that work, treatments that do not work and load management.
- Week 8: Take-home message: What should I do after the treatment? Motivation, habits change, behavioral change and the need to remain active (exercise/treatment).

The mean duration of the patient education videos will be approximately 6 min.

#### Mindfulness group

Participants allocated to this group will receive the same intervention as the control group and an additional pre-recorded video class with MBI content according to their availability (e.g. on Mondays and Wednesdays they receive exercise therapy and Patient education contents; on Fridays they receive mindfulness contents, lasting 35–50 min).

##### Mindfulness-based intervention

The MBI component will be adapted from Mindfulness-Based Health Promotion (MBHP) model to suit patients with PFP and the online assessments of the present study. MBHP is an 8-week MBI developed in 2009 in the context of health promotion and quality of life [60]. Inspired by Jon Kabat-Zinn’s original protocol—mindfulness-based stress reduction, MBSR (University of Massachusetts Medical Center, USA) [61, 62] —it also aggregates elements of other protocols, such as the MBCT (University of Toronto/Canada; University of Cambridge and Oxford University/United Kingdom) [63], the mindfulness programs of the Breathworks Institute (United Kingdom) [64], and mindfulness-based relapse prevention (MBRP, University of Washington, USA [65]. The MBHP has been extensively used in a variety of health conditions [66–68]. Video classes will include formal and informal mindfulness practices. The description of the MBI is available in Additional file 4. The following themes will be covered.

- Week 1: Breaking the automatism.
- Week 2: Body awareness.
- Week 3: Leaving the mind and inhabiting the body.
- Week 4: Raising awareness.
- Week 5: Letting go.
- Week 6: Dealing with challenges and letting go of resistance.
- Week 7: Mindfulness and self-care.
- Week 8: A look to the future.

In addition, participants included in this group will receive daily reminders and additional material (audios) to continue to practice formal and informal MBHP daily. The participants in this group will be encouraged to practice diaries.

### Adherence

In order to improve the adherence to the treatments, before each session, participants of both groups will receive an automatic reminder via SMS and/or email before each session. Participants’ adherence to the interventions will be monitored through the number of accesses (date and hour), time connected to the platform, sessions visualized, number of sessions finalized, number of drop outs and others.

### Adverse events

At the end of each video class, in a pop-up window, all participants will report the intensity of their pain and if there were any adverse events during the session. Participants will be able to contact a therapist through the platform’s email at any time. In case of a severe adverse event related to exercise therapy or MBI (e.g. strains, sprains, persistent severe pain, psychosis, mania, traumatic memories), the participant will be referred to a qualified healthcare professional for further investigation.

### Sample size and power

The sample size calculation was performed based on the usual pain intensity data from Bagheri et al. [30]. Considering a difference between groups of 4.2 mm and a standard deviation of 5.4 mm, with an α of 0.05 and β of 0.20, 26 participants per groups are required (52 in total). We will recruit 31 participants per trial arm to allow for up to a 20% drop-out rate at 12 months.

### Statistical analysis

Statistical analysis will be performed by the blinded assessor using SPSS software (IBM version 23, SPSS Inc., Chicago, Il). Descriptive statistics will be computed for all variables (e.g. mean, standard deviation). Data will be tested for normal distribution by the Shapiro-Wilk test. Chi-square tests will be performed to compare self-reported recovery (successful x unsuccessful) between groups. For continuous data, the effects of group, time and their interaction will be assessed with linear mixed models. Intraclass Correlation Coefficients will be used to determine the amount of variance explained by random effects [69]. The Bonferroni-adjusted *post hoc* test will be performed for multiple pairwise comparisons where appropriate. Effect sizes (95% CI) (Cohen’s d) will also be calculated and interpreted as follows: Cohen’s = 0.2 ‘small effect’; = 0.5 ‘moderate effect’; = 0.8 ‘large effect’ and = 1.3 ‘very large effect’ [70]. Intent-to-treat analyses will be performed for all outcomes. Multiple imputation will be used to account for missing data if the proportion of missing data is > 5% [71]. For all tests, an α level of 0.05 two-tailed will be adopted to indicate statistical significance.

The mediation effects will be assessed following the 3-variable framework described by MacKinnon et al. [72]. In this model, the intervention condition is assumed to have both direct and indirect paths to the changes in clinical outcomes. The indirect path passes through the potential mediators (anxiety, depression, kinesiophobia, pain catastrophizing and pain self-efficacy). Three multiple regressions will be performed: [1] to test the association between the predictor (i.e., interventions) and the outcomes (i.e., pain and function); [2] to test the association between the predictor and the potential mediators and [3] to test the association between the potential mediators and the outcomes after controlling for the predictor. Then, it will be observed whether the association of the predictor with the outcome after controlling for potential mediators will be smaller than observed in the first regression.

## Discussion

PFP is a common and often recalcitrant knee disorder, with symptoms persisting for many years [7]. Exercise therapy and patient education are considered the “best management” options for this population [15, 16]. However, unsatisfactory long-term prognosis remains an issue [17]. It is known that people with PFP present with altered psychological factors [11], which should be considered during the evaluation and treatment of people with PFP [19]. Recent studies suggest that MBI induces functional and structural brain modifications [24, 25]. As part of a rehabilitation program, MBI can help the patients to recognize and accept their condition, promoting a more effective focus on rehabilitation and facilitating pain relief [28, 73]. Therefore, adding a MBI program to the current best treatment for PFP may improve psychological outcomes, providing a better response to treatment at short and long-term. However, this hypothesis needs further investigation. The proposed trial will address this knowledge gap by evaluating the effects of adding an 8-week online MBI program to an online program of exercise therapy and patient education on self-reported recovery, pain, function and psychological factors and in people with PFP. If our hypotheses are confirmed, our findings will contribute to the discussion of a new perspective of treatment modality for people with PFP.

### Limitations

This study investigates the additional effect of an online treatment based on mindfulness program to exercise therapy and patient education for people with PFP. Although exercise therapy and patient education are considered the cornerstones of PFP management [16, 19], the additional effect of the mindfulness intervention to all physical interventions of PFP is not investigated in the present study. Future studies in this area are warranted.

## Electronic supplementary material

Below is the link to the electronic supplementary material.

Supplementary Material 1

Supplementary Material 2

Supplementary Material 3

Supplementary Material 4

Supplementary Material 5

## Data Availability

Not applicable.

## Abbreviations

AKPS: Anterior Knee Pain Scale
CPSS: Chronic Pain Self-Efficacy Scale
GROC: global rating of change scale
HADS: Hospital Anxiety and Depression Scale
IASP: International Association for the Study of Pain
MBHP: Mindfulness-Based Health Promotion
MBI: Mindfulness-based interventions
PCS: Pain Catastrophizing Scale
PFP: Patellofemoral pain
ReBEC: *Registro Brasileiro de Ensaios Clínicos*
SPIRIT: Standard Protocol Items:Recommendations for Interventional Trials
VAS: visual analogue scale.
