# Supplementary material for: Effects of an online program including mindfulness, exercise therapy and patient education compared to online exercise therapy and patient education for people with Patellofemoral Pain: protocol for a randomized clinical trial
Source: BMC Musculoskelet Disord. 2023 May 11;24:372. doi: 10.1186/s12891-023-06491-x (PMC10173555; doi:10.1186/s12891-023-06491-x)
Supplement: Supplementary file 1 — Supplementary Material 1 [file 12891_2023_6491_MOESM1_ESM.docx]

**Additional file 1:** Participant time schedule according to the SPIRIT recommendations.

|  | **STUDY PERIOD** | | | |
| --- | --- | --- | --- | --- |
|  | **Enrolment** | **Allocation** | **Post-allocation** | **Close-out** |
| **TIMEPOINT** | -t1 | t0 | t1 (8 weeks) | t2 (12 months) |
| **ENROLMENT** | | | | |
| Eligibility screen | X |  |  |  |
| Informed consent | X |  |  |  |
| Allocation |  | X |  |  |
| **INTERVENTIONS** | | | | |
| Mindfulness Group |  |  |  |  |
| Control Group |  | 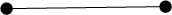 |  |  |
| **ASSESSMENTS:** | | | | |
| Baseline assessment | X | X |  |  |
| Primary and secondary outcomes |  | X | X | X |
