# Supplementary material for: Effects of an online program including mindfulness, exercise therapy and patient education compared to online exercise therapy and patient education for people with Patellofemoral Pain: protocol for a randomized clinical trial
Source: BMC Musculoskelet Disord. 2023 May 11;24:372. doi: 10.1186/s12891-023-06491-x (PMC10173555; doi:10.1186/s12891-023-06491-x)
Supplement: Supplementary file 2 — Supplementary Material 2 [file 12891_2023_6491_MOESM2_ESM.docx]

**Additional file 2.** Details of the interventions according to the TIDieR checklist.

| Brief name: | *Mindfulness Group* | *Located* |
| --- | --- | --- |
| Why? | Adding mindfulness may address psychosocial aspects of PFP and consequently change clinical factors, such as pain and function. | Pages 4 and 5 |
| What (materials)? | Web platform that will provide access to pre-recorded video classes. Materials requested: computer, tablet or notebook to access the web platform; mat or towel to carry out the practice of meditation and exercises; a fixed base to help maintain balance (chair, wall or pillar). | Pages 10 and 11 |
| What (procedures)? | Participants will have access, through login and password, to an online web platform. They will receive the interventions on pre-defined dates organized by the researcher. The intervention will consist of 24 pre-recorded video classes with a similar format to face-to-face care. The video classes will be composed by: (i) Mindfulness-based intervention: formal and informal mindfulness practice; (ii) Exercise therapy: exercises to improve muscle performance, movement coordination and mobility; (iii) Patient education: contents about understanding the knee pain, load management, symptoms and treatment options. | Pages 12 to 14 |
| Who provided? | 2 physiotherapists with more than 3 years of clinical experience will be responsible for creating the exercise and education content. The mindfulness content will be created and recorded by a certificated teacher with more than 15 years of experience and revised by a psychologist to ensure psychological appropriateness. | Page 10 |
| How? | Online, through access to a web platform (available at http://www.stepslab.com.br/) with pre-recorded video classes that will be released in a controlled manner by one researcher not involved in the recruitment and assessment of the participants. | Page 10 |
| Where? | The interventions must be carried out by the participants in their own homes through access to the web platform, via internet. | Page 10 |
| When and how much? | 24 video classes distributed over a period of 8 weeks (2 video classes with exercise and educational contents - lasting 35-50 minutes and 1 video class with mindfulness contents - lasting 30-50 minutes).  Participants in this group will receive extra material to continue to practice formal and informal practices daily. | Page 13 and 14 |
| Tailoring | All participants allocated to this group will receive the same program throughout the intervention period. | Page 10 |
| How well? | The adherence of the participants will be monitored through the number of accesses (date and hour), time connected on the platform, sessions visualized, number of sessions finalized and others. In addition, text messages and emails will be sent before every session to each participant, to remind them to carry out the intervention. | Page 14 |

| Brief name: | *Control Group* |  |
| --- | --- | --- |
| Why? | Currently, the use of exercise-based interventions combined with education is accepted as one of the best available treatments for the management of individuals with PFP. | Pages 3 and 4 |
| What (materials)? | Web platform that will provide access to pre-recorded video classes. Materials requested: computer, tablet or notebook to access the web platform; mat or towel to carry out the exercises; a fixed base to help maintain balance (chair, wall or pillar). | Pages 10 and 11 |
| What (procedures)? | Participants will have access, through login and password, to an online web platform. They will receive the interventions on pre-defined dates organized by the researcher. The intervention will consist of 16 pre-recorded video classes with a similar format to face-to-face care. The video classes will be composed by: (i) Exercise therapy: exercises to improve muscle performance, movement coordination and mobility; (ii) Patient education: contents about understanding the knee pain, load management, symptoms and treatment options. | Pages 12 and 14 |
| Who provided? | 2 physiotherapists with more than 3 years of clinical experience will be responsible for creating the videos contents. | Page 10 |
| How? | Online, through access to a web platform (available at http://www.stepslab.com.br/) with pre-recorded video classes that will be released in a controlled manner by one researcher not involved in the recruitment and evaluation of the participants. | Page 10 |
| Where? | The interventions must be carried out by the participants in their own homes through access to the web platform, via internet. | Page 10 |
| When and how much? | 16 video classes distributed over a period of 8 weeks (2 video classes with exercise and educational contents - lasting 35-50 minutes). | Pages 12 and 13 |
| Tailoring | All participants allocated to this group will receive the same program. | Page 10 |
| How well? | The adherence of the participants will be monitored through the number of accesses (date and hour), time connected on the platform, sessions visualized, number of sessions finished and others. In addition, text messages and emails will be sent before every session to each participant, to remind them to carry out the intervention. | Page 14 |
