# Supplementary material for: Effects of an online program including mindfulness, exercise therapy and patient education compared to online exercise therapy and patient education for people with Patellofemoral Pain: protocol for a randomized clinical trial
Source: BMC Musculoskelet Disord. 2023 May 11;24:372. doi: 10.1186/s12891-023-06491-x (PMC10173555; doi:10.1186/s12891-023-06491-x)
Supplement: Supplementary file 3 — Supplementary Material 3 [file 12891_2023_6491_MOESM3_ESM.docx]

**Additional file 3.** STRUCTURED ONLINE EXERCISE THERAPY FOR PATIENTS WITH PATELLOFEMORAL PAIN

| **PHASE 1** | | |
| --- | --- | --- |
| **Week 1** | | |
| **Session** | **Exercise description** | **Execution guide** |
| **1** | ***STRETCHING***  - Static hamstring stretching  - Static quadriceps stretching  ***STRENGTHENING***  - Isometric anterior plank on knees  - Isometric lateral plank on knees*  - Prone trunk extension  - Side-lying hip abduction with extension and lateral rotation* | ***STRETCHING***  Intensity: Stretch to the point of mild discomfort, stopping before the stretch becomes painful  Sets: 3  Hold time: 30s  Rest in between sets: none (switch limb)  ***STRENGTHENING***  Load: none  Sets: 2  Repetition: 15  Time under tension (con-iso-ecc): 2-0-2s  Hold time: anterior – 30s, lateral – 15s  Rest in between sets: 1 min |
| **2** | ***STRETCHING***  - Static iliotibial band stretching  - Static calf stretching  ***STRENGTHENING***  - Two-legged bridging (floor)  - Donkey kicks (hip extension with lateral rotation) *  - Isometric wall squat (45°)  - Two-legged squats (45°) | ***STRETCHING***  Intensity: Stretch to the point of mild discomfort, stopping before the stretch becomes painful  Sets: 3  Hold time: 30s  Rest in between sets: none (switch limb)  ***STRENGTHENING***  Load: none  Sets: 2  Repetition: 15  Time under tension (con-iso-ecc): 2-0-2s  Hold time: 30s  Rest in between sets: 1 min |
| **Week 2** | | |
| **Session** | **Exercise description** | **Execution guide** |
| **3** | ***STRETCHING***  - Static hamstring stretching  - Static quadriceps stretching  ***STRENGTHENING***  - Isometric anterior plank on knees  - Isometric lateral plank on knees *  - Prone trunk extension  - Side-lying hip abduction with extension and lateral rotation * | ***STRETCHING***  Intensity: Stretch to the point of mild discomfort, stopping before the stretch becomes painful  Sets: 3  Hold time: 30s  Rest in between sets: none (switch limb)  ***STRENGTHENING***  Load: none  Sets: 3  Repetition: 15  Time under tension (con-iso-ecc): 2-0-2s  Hold time: anterior – 30s, lateral – 15s  Rest in between sets: 1 min |
| **4** | ***STRETCHING***  - Static iliotibial band stretching  - Static calf stretching  ***STRENGTHENING***  - Two-legged bridging (floor)  - Donkey kicks (hip extension with lateral rotation) *  - Isometric wall squat (45°)  - Two-legged squats (45°) | ***STRETCHING***  Intensity: Stretch to the point of mild discomfort, stopping before the stretch becomes painful  Sets: 3  Hold time: 30s  Rest in between sets: none (switch limb)  ***STRENGTHENING***  Load: none  Sets: 3  Repetition: 15  Time under tension (con-iso-ecc): 2-0-2s  Hold time: 30s  Rest in between sets: 1 min |

| **PHASE 2** | | |
| --- | --- | --- |
| **Week 3** | | |
| **Session** | **Exercise description** | **Execution guide** |
| **5** | ***STRETCHING***  - Static hamstring stretching  - Static quadriceps stretching  ***STRENGTHENING***  - Isometric anterior plank on feet  - Isometric lateral plank on knees with hip abduction*  - Isometric hip abduction/lateral rotation in standing  - Gluteal crab walk (45°) ** | ***STRETCHING***  Intensity: Stretch to the point of mild discomfort, stopping before the stretch becomes painful  Sets: 3  Hold time: 30s  Rest in between sets: none (switch limb)  ***STRENGTHENING***  Load: none  Sets: 2  Repetition: 12  Time under tension (con-iso-ecc): 2-0-2s  Hold time: anterior – 45s, lateral – 30s  Rest in between sets: 1 min |
| **6** | ***STRETCHING***  - Static iliotibial band stretching  - Static calf stretching  ***STRENGTHENING***  - Two-legged bridging (chair)  - Two-legged bridge with knee extension without arms support  - Isometric wall squat (60°)  - Two-legged squats (90°) | ***STRETCHING***  Intensity: Stretch to the point of mild discomfort, stopping before the stretch becomes painful  Sets: 3  Hold time: 30s  Rest in between sets: none (switch limb)  ***STRENGTHENING***  Load: none  Sets: 2  Repetition: 12  Time under tension (con-iso-ecc): 2-0-2s  Hold time: 1 min  Rest in between sets: 1 min |

| **Week 4** | | |
| --- | --- | --- |
| **Session** | **Exercise description** | **Execution guide** |
| **7** | ***STRETCHING***  - Static hamstring stretching  - Static quadriceps stretching  ***STRENGTHENING***  - Isometric anterior plank on feet  - Isometric lateral plank on knees with hip abduction*  - Isometric hip abduction/lateral rotation in standing  - Gluteal crab walk (45°) ** | ***STRETCHING***  Intensity: Stretch to the point of mild discomfort, stopping before the stretch becomes painful  Sets: 3  Hold time: 30s  Rest in between sets: none (switch limb)  ***STRENGTHENING***  Load: none  Série: 3  Repetition: 12  Time under tension (con-iso-ecc): 2-0-2s  Hold time: anterior – 45s, lateral – 30s  Rest in between sets: 1 min |
| **8** | ***STRETCHING***  - Static iliotibial band stretching  - Static ankle plantar flexors stretching  ***STRENGTHENING***  - Two-legged bridging (chair)  - Two-legged bridge with knee extension without arms support  - Isometric wall squat (60°)  - Two-legged squats (90°) | ***STRETCHING***  Intensity: Stretch to the point of mild discomfort, stopping before the stretch becomes painful  Sets: 3  Hold time: 30s  Rest in between sets: none (switch limb)  ***STRENGTHENING***  Load: none  Sets: 3  Repetition: 12  Time under tension (con-iso-ecc): 2-0-2s  Hold time: 1 min  Rest in between sets: 1 min |

| **PHASE 3** | | |
| --- | --- | --- |
| **Week 5** | | |
| **Session** | **Exercise description** | **Execution guide** |
| **9** | ***WARM-UP***  - Mountain climber  - Jumping jacks  ***STRENGTHENING***  - Isometric anterior plank on feet with alternated hip extension  - Isometric lateral plank on feet*  - Fire hydrant**  - Gluteal crab walk** | ***WARM-UP***  Intensity: maximum speed  Sets: 2  Duration: 1 min  Rest in between sets: 30s  ***STRENGTHENING***  Load: none  Sets: 2  Repetition: 10  Time under tension (con-iso-ecc): 2-2-2s  Hold time: anterior – 60s, lateral – 45s  Rest in between sets: 1 min |
| **10** | ***WARM-UP***  - Jogging in place  - Squat jump  ***STRENGTHENING***  - One-legged bridging (floor) *  - Isometric wall squat (90°)  - One-legged bridge with knee extension with arms support **  - Sit to stand (One/two-legged) * | ***WARM-UP***  Intensity: maximum speed  Sets: 2  Duration: 1 min  Rest in between sets: 30s  ***STRENGTHENING***  Load: none  Sets: 2  Repetition: 10  Time under tension (con-iso-ecc): 2-2-2s  Hold time: 1 min  Rest in between sets: 1 min |

| **Week 6** | | |
| --- | --- | --- |
| **Session** | **Exercise description** | **Execution guide** |
| **11** | ***WARM-UP***  - Mountain climber  - Jumping jacks  ***STRENGTHENING***  - Isometric anterior plank on feet with alternated hip extension  - Isometric lateral plank on feet *  - Fire hydrant *  - Gluteal crab walk ** | ***WARM-UP***  Intensity: maximum speed  Sets: 2  Duration: 1 min  Rest in between sets: 30s  ***STRENGTHENING***  Load: none  Sets: 3  Repetition: 10  Time under tension (con-iso-ecc): 2-2-2s  Hold time: anterior – 60s, lateral – 45s  Rest in between sets: 1 min |
| **12** | ***WARM-UP***  - Jogging in place  - Squat jump  ***STRENGTHENING***  - One-legged bridging (floor) *  - Isometric wall squat (90°)  - One-legged bridge with knee extension with arms support **  - Sit to stand (One/two-legged) * | ***WARM-UP***  Intensity: maximum speed  Sets: 2  Duration: 1 min  Rest in between sets: 30s  ***STRENGTHENING***  Load: none  Sets: 3  Repetition: 10  Time under tension (con-iso-ecc): 2-2-2s  Hold time: 1 min  Rest in between sets: 1 min |

| **PHASE 4** | | |
| --- | --- | --- |
| **Week 7** | | |
| **Session** | **Exercise description** | **Execution guide** |
| **13** | ***WARM-UP***  - High knees  - Burpee  ***STRENGTHENING***  - Superman  - Isometric anterior plank on feet with hip shift  - Isometric lateral plank on feet **  - Forward lunges ** | ***WARM-UP***  Intensity: maximum speed  Sets: 2  Duration: 1 min  Rest in between sets: 30s  ***STRENGTHENING***  Load: none  Sets: 2  Repetition: 8  Time under tension (con-iso-ecc): 2-2-2s  Hold time: anterior – 75s, lateral – 60s  Rest in between sets: 1 min |
| **14** | ***WARM-UP***  - Squat with jumping jacks  - Leaping lateral squat  ***STRENGTHENING***  - One-legged bridging (chair) **  - Isometric wall squat (90°)  - One-legged sit to stand *  - One-legged bridge with knee extension without arms support ** | ***WARM-UP***  Intensity: maximum speed  Sets: 2  Duration: 1 min  Rest in between sets: 30s  ***STRENGTHENING***  Load: none  Sets: 2  Repetition: 8  Time under tension (con-iso-ecc): 2-2-2s  Hold time: 1 min  Rest in between sets: 1 min |

| **Week 8** | | |
| --- | --- | --- |
|  | **Exercise description** | **Execution guide** |
| **15** | ***WARM-UP***  - High knees  - Burpee  ***STRENGTHENING***  - Superman  - Isometric anterior plank on feet with hip shift  - Isometric lateral plank on feet with hip abduction **  - Forward lunges ** | ***WARM-UP***  Intensity: maximum speed  Sets: 2  Duration: 1 min  Rest in between sets: 30s  ***STRENGTHENING***  Load: none  Sets: 3  Repetition: 8  Time under tension (con-iso-ecc): 2-2-2s  Hold time: anterior – 75s, lateral – 60s  Rest in between sets: 1 min |
| **16** | ***WARM-UP***  - Jumping lunges  - Leaping lateral squat  ***STRENGTHENING***  - One-legged bridging (chair) *  - Isometric wall squat (90°)  - One-legged sit to stand *  - One-legged bridge with knee extension without arms support ** | ***WARM-UP***  Intensity: maximum speed  Sets: 2  Duration: 1 min  Rest in between sets: 30s  ***STRENGTHENING***  Load: none  Sets: 3  Repetitions: 8  Time under tension (con-iso-ecc): 2-2-2s  Hold time: 1 min  Rest in between sets: 1 min |

Footnotes: The “Structured online exercise therapy for patients with patellofemoral pain” aims to promote pain relief, improvements of lower limbs and trunk mobility and strength through exercise progression. This was made based on intensity and difficulty increases.

(con-iso-ecc), concentric-isometric-eccentric.

* One-legged exercises: no rest between sets – perform the exercise with alternating limbs. Rest only at the end of the exercise.

** One-legged exercises: rest between sets.
