# Supplementary material for: Effects of an online program including mindfulness, exercise therapy and patient education compared to online exercise therapy and patient education for people with Patellofemoral Pain: protocol for a randomized clinical trial
Source: BMC Musculoskelet Disord. 2023 May 11;24:372. doi: 10.1186/s12891-023-06491-x (PMC10173555; doi:10.1186/s12891-023-06491-x)
Supplement: Supplementary file 4 — Supplementary Material 4 [file 12891_2023_6491_MOESM4_ESM.docx]

**Additional file 4.** ONLINE MINDFULNESS-BASED INTERVENTION FOR PATIENTS WITH PATELLOFEMORAL PAIN

| **WEEK** | **TEACHING CONTENT** | **PRACTICES** | **HOMEWORK** |
| --- | --- | --- | --- |
| **1. Breaking the automatism** | - Presentation and aims  - Formal and informal practices  - What is and what is not mindfulness - Attention-intention attitude  - Motivation to practice  - Orientations and precautions to practice  - Meditation postures  - Practice journaling | - Mindful eating: the raisin exercise  - Mindfulness of the body and breath  - Commitment letter | **Formal:**  - Mindfulness of the body and breath (10 to 12min)  **Informal:**  - Mindfulness during everyday activities: choose one daily activity to practice mindfully (e.g., eating one meal mindfully)  - Practice journaling |
| **2. Body awareness** | - Barriers and facilitators to the mindfulness practice  - “Primary” and “secondary” suffering  - Importance of body awareness  - Mindful movements  - Awareness of pleasant events (journaling) | - Body scan  - Primary and secondary suffering (experiencing the physical discomfort/ knee pain)  - Mindful walking | **Formal:**  - Body scan  **Informal:**  - Mindfulness during everyday activities (e.g., casual walking)  - Awareness of pleasant events (journalingdiary)  - Lifestyle behavior change (choose a small lifestyle habit to change) |
| **3. Leaving the mind and inhabiting the body** | - Focusing attention on breathing  - Didactic exploration of breathing  - Distracted mind vs attentive mind  - Dealing with thoughts  - Mindfulness attitude  - Awareness of unpleasant events (journaling) | - Mindful breathing  - Hello, thank you and goodbye exercise | **Formal:**  - Mindful breathing/ Body scan (alternate days)  **Informal:**  - Mindfulness during everyday activities (e.g., brushing teeth with the non-dominant hand)  - Awareness of unpleasant events ( journaling)  - Lifestyle behavior change (choose a small lifestyle habit to change) |
| **4. Raising awareness** | - The importance of regular practice  - Negativity bias  - Attachment x aversion | - Mindfulness in breathing, sensations, sounds and thoughts and open monitoring  - 3-minutes practice  - Mindful movements | **Formal:**  - Mindfulness in breathing, sensations, sounds and thoughts and open monitoring  - Mindful movements (stretching)  **Informal:**  - Mindfulness during everyday activities  - 3-minutes practice (2 times/ day)  - Lifestyle behavior change (choose a small lifestyle habit to change) |
| **5. Letting go** | - What to do with the mind  - Cognitive strategies to deal with thoughts  - Thoughts are not facts  - Doing mode/ being mode | - Mindfulness in breathing, sensations, sounds and thoughts with labelling and metaphors | **Formal:**  - Mindfulness in breathing, sensations, sounds and thoughts with labelling and metaphors  - 3-minutes practice (2 times/ day)  **Informal:**  - Mindfulness during everyday activities  - Mindful movements (stretching)  - Lifestyle behavior change (choose a small lifestyle habit to change) |
| **6. Dealing with challenges and letting go of resistance** | - Acceptance attitude  - Attitude of letting go vs resisting  - The stages of acceptance; care actions  - Giving permission to just let the experience be however it is (unpleasant/pleasant/neutral)  - The importance of breathing pauses in challenging situations  - Stressors and response to stressors  - Strategies to use while experiencing difficult emotions | - Recognizing emotions  - Pleasant and unpleasant meditation | **Formal:**  - Pleasant and unpleasant meditation/ Mindfulness in breathing, sensations, sounds, thoughts and emotions (alternate days)  **Informal:**  - 3-minutes practice during challenging situations  - Practice of choice in routine activity  - Lifestyle behavior change (choose a small lifestyle habit to change) |
| **7. Mindfulness and self-care** | -What is and what is not compassion, empathy, resignation and indulgence  -Physiological experience of compassion  -Biological foundations of compassion  -Self-compassion and our bodies  - Identifying our essential values  - How can I take care of myself?  - Nourishing vs depleting activities | - Self-compassion  - Self-compassion break  - Exercise: Discovering our essential values  - Nourishing and depleting activities worksheet | **Formal:**  - Pleasant and unpleasant meditation/ Self-compassion (alternate days)  - Self-compassion break in moments of pain/ challenges  **Informal:**  - Practice of choice in routine activity  - Action plan |
| **8. A look to the future** | - Awareness of interdependence  - Compassion and emotional resilience  - Cultivating mindfulness in everyday life  - Preparing for the future - How can I continue to care for myself?  - Personal reflections and commitment to self-care practices | - Compassionate body scan  - Loving-kindness (for oneself and others)  - Putting the action plan into practice | **Formal:**  - Compassionate body scan  - Loving-kindness (for oneself and others)  **Informal:**  **-** Action plan  - Practice of choice in routine activity  - Mindfulness during everyday activities |
